# Navigating sexual health concerns in the medical profession: A Tunisian perspective

**DOI:** 10.1192/j.eurpsy.2025.2310

**Published:** 2025-08-26

**Authors:** A. Karmous, H. Ghabi, N. Karmous, E. Khelifa, A. Maamri, H. Zalila

**Affiliations:** 1 emergency and outpatient department; 2 Department G, Razi hospital, Manouba; 3 Gynaecology and obstetrics department B, Charles Nicoles Hospital, Tunis, Tunisia

## Abstract

**Introduction:**

Sexual functioning is an essential aspect of human existence. Sexual dysfunctions are prevalent and negatively affect the quality of life in the general population. Little attention has been paid to the sexual function of health workers.

**Objectives:**

The aim of our work was to evaluate sexual dysfunction among Tunisian doctors and to determine associated underlying factors.

**Methods:**

A cross-sectional study was conducted online, from January to March 2024, via a pre-established questionnaire. Tunisian doctors, who had finished their medical studies, working in the public or private sector and who agreed to anonymously respond to the questionnaire were included. Sociodemographic, economic, clinical data and those related to the medical profession were collected. Sexual function was evaluated with the Arizona Sexual Experience Scale (ASEX).

**Results:**

A total of 80 individuals had fully responded to the questionnaire. The mean age of participants was 36.81 ± 7.49 years and 68,7 % (n=55) of them were male. Ninety five percent (n=76) were married and 77.5 % (n=62) had children. The mean working hours per week was 34.32 ± 5.32. A regular physical activity was practiced by 35 % (n=28) of individuals. The mean monthly income was 3592.5 ± 596 Tunisian Dinars. The results of the ASEX showed that 23,8 % (n=19) of participants, 23,6 % (n=13) of male participants and 24 % of female (n=6) participants had sexual dysfunction. Ten per cent of individuals (n=8) have previously consulted a sexologist. Longer working hours, lower monthly income, less physical exercise were significantly associated with increased risk for sexual dysfunction.

**Conclusions:**

Sexual dysfunctions seem to be common among Tunisian doctors. Knowledge about their sexual functioning is important to promote their physical and mental health and to improve the care delivered.

**Disclosure of Interest:**

None Declared

